# Harmonization of Molecular Testing for Non-Small Cell Lung Cancer: Emphasis on PD-L1

**DOI:** 10.3389/fonc.2020.549198

**Published:** 2020-09-30

**Authors:** Evgeny N. Imyanitov, Alexandr O. Ivantsov, Ilya V. Tsimafeyeu

**Affiliations:** ^1^Department of Tumor Growth Biology, N.N. Petrov Institute of Oncology, St. Petersburg, Russia; ^2^Department of Clinical Genetics, St.-Petersburg Pediatric Medical University, Saint Petersburg, Russia; ^3^Institute of Oncology, Hadassah Moscow, Moscow, Russia

**Keywords:** non-small cell lung cancer, molecular testing, PD-L1, PCR, review

## Abstract

Comprehensive molecular testing plays a critical role in the choice of treatment for non-small lung cell cancer (NSCLC). The analysis of druggable alterations in EGFR, BRAF, MET, KRAS, ALK, ROS1, RET and NTRK1/2/3 genes is more or less standardized and can be achieved using a single diagnostic platform, e.g., next generation sequencing (NGS) or polymerase chain reaction (PCR). In contrast to above targets, PD-L1 testing requires the use of immunohistochemistry (IHC). There are multiple PD-L1 IHC assays, which utilize distinct antibodies and detection systems. These PD-L1 tests are tailored to distinct drugs, often rely on different thresholds and scoring guidelines, and are characterized by incomplete inter-laboratory and inter-observer reproducibility. Several studies evaluated the performance of PD-L1 RNA expression tests, as PCR-based RNA analysis is compatible with other NSCLC molecular testing platforms, can be performed in a semi-automated manner, and has a potential for proper standardization. These investigations revealed a correlation between PD-L1 protein and RNA expression; however, there were NSCLCs demonstrating decent amounts of PD-L1 transcript in the absence of PD-L1 IHC staining. Clinical studies are required to evaluate, which of the two PD-L1 testing approaches, i.e., RNA or protein expression measurement, has a better predictive value.

While only a decade ago the laboratory diagnosis of non-small cell lung cancer (NSCLC) required mainly conventional morphological analysis, the process of examination of NSCLC tissues is getting increasingly complex nowadays, thanks to the invention of new targeted drugs. EGFR tyrosine kinase inhibitors (TKIs) were the first to trigger the molecular profiling of lung cancer, as they demonstrated high response rates in tumors with EGFR exon 19 and 21 drug-sensitizing mutations. Subsequent advances were based on the discovery of ALK and ROS1 rearrangements, which also turned to be linked to the pronounced tumor sensitivity to corresponding TKIs. Interestingly, the development of gefitinib, erlotinib and crizotinib actually preceded the identification of their genuine molecular targets, so the incorporation of these drugs into the NSCLC management was somehow attributed to some chance discoveries. This is in stark contrast with the history of the invention of inhibitors of the mutated BRAF, which is clearly an output of a pre-planned research, starting from the systematic search for kinase activating mutations and eventually resulting in the intentional development of specific antagonists of the BRAF V600E protein. There is a multitude of new NSCLC drug targets, e.g., NTRK1-3 and RET gene fusions, MET exon 14 skipping mutations, KRAS G12C substitutions, etc. In addition, administration of several inhibitors of immune checkpoints involves testing for PD-L1 expression (1–3).

NSCLC diagnostic pipeline includes a spectrum of molecular assays which usually rely on distinct laboratory platforms. The analysis of EGFR, BRAF, and KRAS mutations usually requires allele-specific PCR and/or gene sequencing. The detection of ALK, ROS, RET, and NTRK1-3 rearrangements may be based on the immunohistochemistry (IHC) guided detection of the overexpression of the kinase portion of the corresponding protein or on the break-apart FISH assay (3). The methodology of MET testing remains to be standardized (4). PD-L1 expression analysis is apparently the most complicated assay for the time being. There are several approved antibodies for the PD-L1 status evaluation. These antibodies are tailored to particular diagnostic platforms, linked to the use of distinct therapeutic modulators of the PD-L1/PD1 pathway, have different scoring guidelines and utilize varying thresholds between “positive” and “negative” samples. The detailed listing of PD-L1 antibodies, detection systems, associated therapeutic compounds and staining patterns is provided in several reviews (5–7). Most importantly, while the majority of clinical trials involving PD-L1/PD1 pathway inhibitors generally demonstrate an association between PD-L1 expression and clinical benefit from the drug, there is a great variability across the NSCLC studies with regard to medical applicability of observed findings (Table 1).

**Table 1:** Selected clinical studies on immune checkpoint inhibitors, which evaluated associations between clinical outcomes and the level of PD-L1 expression analysis.

**References**	**Brief description of the study**	**Survival**	**Predictive role of PD-L1**
**Pembrolizumab (IHC: 22C3)**			
Herbst et al. (8) (KEYNOTE-010)	Assessment of long-term outcomes of pembrolizumab vs. docetaxel monotherapy in previously treated NSCLC with PD-L1 expression in >/=1% tumor cells	OS PD-L1 1-49%: pembrolizumab: 11.8 months; docetaxel: 8.4 months PD-L1 >/=50%: pembrolizumab: 16.9 months; docetaxel: 8.2 months	Dramatic improvement of OS for pembrolizumab in patients with PD-L1 expression in >/=50% tumor cells; moderate improvement of OS in patients with PD-L1 expression score 1–49%
Gadgeel et al. (9) (KEYNOTE-189)	First-line therapy, non-squamous NSCLC: pembrolizumab or placebo plus pemetrexed and platinum	OS PD-L1 <1%: pembrolizumab plus chemotherapy: 17.2 months; pembrolizumab plus placebo: 10.2 months PD-L1 1-49%: pembrolizumab plus chemotherapy: 21.8 months; pembrolizumab plus placebo: 12.1 months PD-L1 >/=50%: pembrolizumab plus chemotherapy: not reached; pembrolizumab plus placebo: 10.1 months PFS PD-L1 <1%: pembrolizumab plus chemotherapy: 6.2 months; pembrolizumab plus placebo: 5.1 months PD-L1 1-49%: pembrolizumab plus chemotherapy: 9.2 months; pembrolizumab plus placebo: 4.9 months PD-L1 >/=50%: pembrolizumab plus chemotherapy: 11.1 months; pembrolizumab plus placebo: 4.8 months	Pembrolizumab plus chemotherapy outperformed pembrolizumab plus placebo regardless of the PD-L1 status, however the magnitude of the effect was higher in tumors with high PD-L1 expression
Garon et al. (10) (KEYNOTE-001)	Assessment of long-term outcomes of pembrolizumab monotherapy in treatment-naïve and previously treated patients	OS in treatment-naïve patients: PD-L1 <1%: not evaluated (low number of patients) PD-L1 1–49%: 19.5 months PD-L1 >/=50%: 35.4 months OS in previously treated patients: PD-L1 <1%: 8.6 months PD-L1 1–49%: 8.5 months PD-L1 >/=50%: 15.4 months	Pembrolizumab treatment was associated with improved OS in patients with PD-L1 expression in >/=50% tumor cells
Mok et al. (11) (KEYNOTE-042)	First-line therapy: pembrolizumab vs. chemotherapy for NSCLC with PD-L1 expression in >/=1% tumor cells	OS: PD-L1 >/=1%: pembrolizumab: 16.7 months; chemotherapy: 12.1 months PD-L1 >/=20%: pembrolizumab: 17.7 months; chemotherapy: 13.0 months PD-L1 >/=50%: pembrolizumab: 20.0 months; chemotherapy: 12.2 months PFS: PD-L1 >/=1%: pembrolizumab: 5.4 months; chemotherapy: 6.5 months PD-L1 >/=20%: pembrolizumab: 6.2 months; chemotherapy: 6.6 months PD-L1 >/=50%: pembrolizumab: 7.1 months; chemotherapy: 6.4 months	Improvement of OS for pembrolizumab was observed both for PD-L1 >/=50% and >/=1% expression thresholds, however the magnitude of the effect was greater for the high expressors
Paz-Ares et al. (12) (KEYNOTE-407)	First-line therapy, squamous NSCLC: pembrolizumab or placebo plus chemotherapy	OS: PD-L1 <1%: pembrolizumab plus chemotherapy: 15.9 months; pembrolizumab plus placebo: 10.2 months PD-L1 1–49%: pembrolizumab plus chemotherapy: 14.0 months; pembrolizumab plus placebo: 11.6 months PD-L1 >/=50%: pembrolizumab plus chemotherapy: not reached; pembrolizumab plus placebo: not reached PFS: PD-L1 <1%: pembrolizumab plus chemotherapy: 6.3 months; pembrolizumab plus placebo: 5.3 months PD-L1 1–49%: pembrolizumab plus chemotherapy: 7.5 months; pembrolizumab plus placebo: 5.2 months PD-L1 >/=50%: pembrolizumab plus chemotherapy: 8.0 months; pembrolizumab plus placebo: 4.2 months	Pembrolizumab plus chemotherapy outperformed pembrolizumab plus placebo regardless of the PD-L1 status
**Nivolumab (IHC: 28-8)**			
Hellmann et al. (13) (CHECKMATE 227)	First-line therapy: nivolumab plus ipilimumab vs. nivolumab alone vs. chemotherapy for NSCLC with PD-L1 expression in >/=1% tumor cells; nivolumab plus ipilimumab vs. nivolumab plus chemotherapy vs. chemotherapy for NSCLC with PD-L1 expression in <1% tumor cells	OS: PD-L1 <1%: nivolumab plus ipilimumab: 17.2 months; nivolumab plus chemotherapy: 15.2 months; chemotherapy: 12.2 months PD-L1 >/=1%: nivolumab plus ipilimumab: 17.1 months; nivolumab alone: 15.7 months; chemotherapy: 14.9 months PD-L1 >/=50%: nivolumab plus ipilimumab: 21.2 months; nivolumab alone: 18.1 months; chemotherapy: 14.0 months PFS: PD-L1 <1%: nivolumab plus ipilimumab: 5.1 months; nivolumab alone: 5.6 months; chemotherapy: 4.7 months PD-L1 >/=1%: nivolumab plus ipilimumab: 5.1 months; nivolumab alone: 4.2 months; chemotherapy: 5.6 months PD-L1 >/=50%: nivolumab plus ipilimumab: 6.7 months; nivolumab alone: 5.6 months; chemotherapy: 5.6 months	Nivolumab plus ipilimumab outperformed chemotherapy regardless of the PD-L1 status, however the difference was more pronounced in patients with PD-L1 expression in >/=50% tumor cells
Ready et al. (14) (CHECKMATE 568)	First-line therapy: nivolumab plus ipilimumab	PFS: PD-L1 <1%: 2.8 months PD-L1 >/=1%: 6.8 months PD-L1 >/=50%: 6.8 months	PD-L1 expression was associated with higher rate of objective responses and longer PFS
Carbone et al. (15) (CHECKMATE 026)	First-line therapy: nivolumab vs. chemotherapy for NSCLC with PD-L1 expression in >/=1% tumor cells	OS: PD-L1 >/=1%: nivolumab: 13.7 months; chemotherapy: 13.8 months PD-L1 >/=5%: nivolumab: 14.4 months; chemotherapy: 13.2 months PD-L1 >/=50%: nivolumab: 15.9 months; chemotherapy: 13.9 months PFS: PD-L1 >/=1%: nivolumab: 4.2 months; chemotherapy: 5.8 months PD-L1 >/=5%: nivolumab: 4.2 months; chemotherapy: 5.9 months PD-L1 >/=50%: nivolumab: 5.4 months; chemotherapy: 5.8 months	No predictive value for PD-L1 expression
Borghaei et al. (16) (CHECKMATE 057)	Nivolumab vs. docetaxel monotherapy in previously treated patients with non-squamous NSCLC	OS: PD-L1 <1%: nivolumab: 10.5 months; docetaxel: 10.1 months PD-L1 >/=1%: nivolumab: 17.7 months; docetaxel: 9.0 months PD-L1 >/=5%: nivolumab: 19.4 months; docetaxel: 8.1 months PD-L1 >/=10%: nivolumab: 19.9 months; docetaxel: 8.0 months PFS: PD-L1 <1%: nivolumab: 2.1 months; docetaxel: 3.6 months PD-L1 >/=1%: nivolumab: 4.2 months; docetaxel: 4.5 months PD-L1 >/=5%: nivolumab: 5.0 months; docetaxel: 3.8 months PD-L1 >/=10%: nivolumab: 5.0 months; docetaxel: 3.7 months	Nivolumab outperformed docetaxel only in patients with PD-L1 expression in >/=1% tumor cells
Brahmer et al. (17) (CHECKMATE 017)	Nivolumab vs. docetaxel monotherapy in previously treated patients with squamous NSCLC	OS: PD-L1 <1%: nivolumab: 8.7 months; docetaxel: 5.9 months PD-L1 >/=1%: nivolumab: 9.3 months; docetaxel: 7.2 months PD-L1 >/=5%: nivolumab: 10.0 months; docetaxel: 6.4 months PD-L1 >/=10%: nivolumab: 11.0 months; docetaxel: 7.1 months	No predictive role of the PD-L1 status
		PFS: PD-L1 <1%: nivolumab: 3.1 months; docetaxel: 3.0 months PD-L1 >/=1%: nivolumab: 3.3 months; docetaxel: 2.8 months PD-L1 >/=5%: nivolumab: 4.8 months; docetaxel: 3.1 months PD-L1 >/=10%: nivolumab: 3.7 months; docetaxel: 3.3 months	
**Atezolizumab (IHC: SP142)**			
Socinski et al. (18) (IMpower 150)	First-line therapy: atezolizumab plus carboplatin plus paclitaxel (ACP) vs. bevacizumab plus carboplatin plus paclitaxel (BCP) vs. atezolizumab plus BCP (ABCP) for non-squamous NSCLC	ABCP vs. BCP comparison, PFS: TC3 or IC3: 12.6 months vs. 6.8 months TC1/2/3 or IC1/2/3: 11.0 months vs. 6.8 months TC1/2 or IC1/2: 8.3 months vs. 6.6 months TC0/1/2 and IC0/1/2: 8.0 months vs. 6.8 months TC0 and IC0: 7.1 months vs. 6.9 months	Addition of atezolizumab to carboplatin, paclitaxel and bevacizumab improved PFS regardless of the PD-L1 status, however the magnitude of the effect was higher for tumors with high PD-L1 expression
Rittmeyer et al. (19) (OAK)	Atezolizumab vs. docetaxel monotherapy in previously treated NSCLC patients	OS: TC3 or IC3: 20.5 months vs. 8.9 months TC2/3 or IC2/3: 16.3 months vs. 10.8 months TC1/2/3 or IC1/2/3: 15.7 months vs. 10.3 months TC0 and IC0: 12.6 months vs. 8.9 months	Atezolizumab outperformed docetaxel regardless of the PD-L1 status, however the magnitude of the effect was higher for tumors with high PD-L1 expression
Fehrenbacher et al. (20) (POPLAR)	Atezolizumab vs. docetaxel monotherapy in previously treated NSCLC patients	OS: TC3 or IC3: 15.5 months vs. 11.1 months TC2/3 or IC2/3: 15.1 months vs. 7.4 months TC1/2/3 or IC1/2/3: 15.5 months vs. 9.2 months TC0 and IC0: 9.7 months vs. 9.7 months	Atezolizumab outperformed docetaxel only in patients with PD-L1 expression in >/=1% tumor cells or >/=1% tumor-infiltrating immune cells
**Avelumab: (IHC: 73-10)**			
Barlesi et al. (21) (JAVELIN Lung 200)	Avelumab vs. docetaxel monotherapy in previously treated NSCLC patients	OS: PD-L1 >/=1%: avelumab: 11.4 months; docetaxel: 10.3 months PD-L1 >/=50%: avelumab: 13.6 months; docetaxel: 9.2 months PD-L1 >/=80%: avelumab: 17.1 months; docetaxel: 9.3 months	Improved outcomes for avelumab were observed only in patients with high PD-L1 expression
Gulley et al. (22) (JAVELIN Solid Tumor)	Avelumab in previously treated NSCLC patients	OS: PD-L1 <1% tumor cells: 4.6 months PD-L1 >/=1% tumor cells: 8.9 months PD-L1 >/=5% tumor cells: 10.6 months PD-L1 >/=25% tumor cells: 8.4 months PD-L1 </=10% immune cells in hot-spots: 8.5 months PD-L1 >/=10% immune cells in hot-spots: 8.9 months PFS: PD-L1 <1% tumor cells: 5.9 weeks PD-L1 >/=1% tumor cells: 12.0 weeks PD-L1 >/=5% tumor cells: 11.9 weeks PD-L1 >/=25% tumor cells: 11.9 weeks PD-L1 </=10% immune cells in hot-spots: 11.3 weeks PD-L1 >/=10% immune cells in hot-spots: 8.4 weeks	Improved outcomes for avelumab were observed when PD-L1 expression in >/=1% tumor cells was used as a threshold
**Durvalumab (IHC: SP263)**			
Rizvi et al. (23) (MYSTIC)	First-line therapy: durvalumab with or without tremelimumab vs. standard chemotherapy	OS: PD-L1 <1%: durvalumab plus tremelimumab: 11.9 months; durvalumab alone: 10.1 months; chemotherapy: 10.3 months PD-L1 >/=1%: durvalumab plus tremelimumab: 10.9 months; durvalumab alone: 14.6 months; chemotherapy: 12.3 months PD-L1 25–49%: durvalumab plus tremelimumab: 10.5 months; durvalumab alone: 11.1 months; chemotherapy: 13.3 months PD-L1 >/=50%: durvalumab plus tremelimumab: 15.2 months; durvalumab alone: 18.3 months; chemotherapy: 12.7 months	Improved outcomes for durvalumab were observed only in patients with high PD-L1 expression
Paz-Ares et al. (24) (PACIFIC)	Durvalumab vs. placebo after chemoradiotherapy in unresectable stage III NSCLC	OS: PD-L1 <1%: durvalumab 33.1 months; placebo: 45.6 months PD-L1 1–24%: durvalumab 43.3 months; placebo: 30.5 months PD-L1 >/=25%: durvalumab: not reached; placebo: 21.1 months PFS: PD-L1 <1%: durvalumab 10.7 months; placebo: 5.6 months PD-L1 1–24%: durvalumab: not reached; placebo: 9.0 months PD-L1 >/=25%: durvalumab 17.8 months; placebo: 3.7 months	Improved PFS for durvalumab was observed across all subgroups; improved OS for durvalumab was seen for patients with PD-L1 expression in >/=1% tumor cells
Garassino et al. (25) (ATLANTIC)	Durvalumab as a third-line or later treatment in NSCLC patients	OS: Cohort EGFR+/ALK+: PD-L1 <25%: 9.9 months PD-L1 >/=25%: 13.3 months Cohort EGFR-/ALK-: PD-L1 <25%: 9.3 months PD-L1 >/=25%: 10.9 months Cohort PD-L1>/=90%: not reached PFS: Cohort EGFR+/ALK+: PD-L1 <25%: 1.9 months PD-L1 >/=25%: 1.9 months Cohort EGFR-/ALK-: PD-L1 <25%: 1.9 months PD-L1 >/=25%: 3.3 months Cohort PD-L1>/=90%: 2.4 months	PD-L1 expression was associated with improved outcomes

*OS, overall survival; PFS, progression-free survival; IHC, immunohistochemistiry; TC, tumor cells; IC, immune cells; the details for TC and IC scoring are explained in (20)*.

Many NSCLCs are diagnosed as a metastatic disease, therefore tumor tissue material is represented by a single tiny biopsy sample. These samples must be divided for mutational analysis (PCR, sequencing) and visualization-based tests (IHC, FISH). There is a great need for a “one-for-all” approach, which would allow for a comprehensive NSCLC examination performed on a single platform. Next generation sequencing (NGS) provides a viable diagnostic opportunity, as it is capable of detecting all relevant genetic alterations within a single run. At the present time, NGS has significant limitations, such as relatively high cost, need for significant turn-around time, and requirement for sophisticated equipment (3, 26, 27). Furthermore, NGS is not yet fully compatible with a high-precision analysis of gene expression. There are ongoing efforts to utilize PCR for all types of NSCLC molecular analysis. These assays include simultaneous isolation of DNA and RNA in a single tube, synthesis of complementary DNA (cDNA) on the RNA template, conventional analysis of mutations and the test for 5′/3′-end unbalanced expression of rearranged kinases. The latter approach allows for identification of all druggable gene fusions irrespective of the translocation variant (28). PCR analysis is relatively non-expensive and is more flexible for the incorporation of new predictive tests, as exemplified by the development of the assay for detection of MET exon 14 skipping mutations (4).

While many NSCLC tests can be performed by a number of interchangeable approaches, PD-L1 analysis remains restricted to IHC technology. Use of IHC scoring is time-consuming and may be a subject of significant interobserver variability (5–7). Explicit analysis of the comparability of the existing IHC assays has been recently published by Koomen et al. (29). PD-L1 IHC comparative studies generally demonstrate acceptable results with regard to assays' interchangeability, inter-observer variability, and inter-laboratory agreement. However, it is necessary to keep in mind that the pathologists involved in research activities and scientific publishing are likely to have somewhat better standards of the laboratory practice, so the real-world inconsistencies in the IHC performance may be substantially higher when compared to pre-planned investigations. In addition, while the numerical comparisons of PD-L1 scores show good correlation, there is an alarming rate of discordance when clinically accepted thresholds are utilized (29). Consider a situation in which one pathologist determines the proportion of PD-L1 tumor cells slightly below 1%, while another pathologist determines this proportion to be slightly over 1%. When formal correlation coefficients for continuous numerical variables are calculated, these two results will be considered concordant; however, in clinical practice this difference may critically affect the access to immune therapy, as PD-L1 score of 1% is a commonly accepted threshold for consideration of immune therapies in several clinical scenarios.

Measurement of RNA expression of the gene of interest can offer advantages over IHC. In particular, PCR-based RNA expression analysis offers better reproducibility, as it evaluates not the quantity of the gene-specific transcript *per se*, but the ratio between the RNA messages of the gene-target and gene-referee. Furthermore, PCR tests are usually performed in a semi-automated manner, so they are less labor-consuming as compared to morphology-based analyses (30–32). However, RNA testing has several limitations. First, gene transcription is not always an equivalent of gene translation, as the production and decay of gene-specific RNAs and proteins involves different layers of regulation. Second, IHC analysis is capable to assess intracellular localization of the protein, while RNA assays evaluate only the bulk amount of gene product. Third, some analytical solutions, such as PD-L1 IHC assays, offer individual scoring for various cell types, for example, tumor cells and immune cells (6, 7). This advantage is not compatible with currently established PCR procedures. Several studies attempted to investigate in parallel the expression of PD-L1 on the level of RNA and protein in cell cultures and tumor tissues. These small-scale studies provided generally encouraging results indicating that the correlation between PD-L1 RNA and protein level does exist (30–36).

Recently published CLOVER study represents the first systematic attempt to evaluate the feasibility of PCR-based PD-L1 testing in comparison with IHC (37). The authors analyzed 437 NSCLC samples by three PD-L1 IHC assays (Ventana SP142, Ventana SP263, Dako 22C3) and by the laboratory-developed real-time PCR test for PD-L1 RNA expression. In agreement with other investigations, the CLOVER study showed significant concordance between the SP263 and the 22C3 IHC, while the SP142 produced lower rate of PD-L1 positive tumors. Indeed, the Blueprint Phase 1 study, which included 39 lung tumors stained with four different antibodies, showed that SP263 and 22C3 assays demonstrated similar IHC patterns in the majority of cases, while SP142 stained fewer number of tumor cells with generally lower intensity (38). The Blueprint Phase 2 study essentially replicated these results using a “real-world” series of 81 lung cancer specimens (39). The CLOVER study compared the performance of PCR-based PD-L1 expression measurement against conventional IHC assays. Strikingly, negative PCR tests appeared to be a reliable predictor for the lack of PD-L1 expression as determined by immunohistochemistry. This is an expected observation, given that the PCR is considered to be an ultrasensitive method for detection of biological molecules; so if the gene product cannot be detected by PCR it is unlikely to be seen by other methods. However, positive predictive value of PCR was low, as many PD-L1 RNA expressing tumors turned out to be PD-L1-negative by IHC analysis.

The results of the CLOVER study may potentially be relevant to already existing PCR diagnostic pipelines. It is relatively easy to add one more gene-specific assay to established PCR procedures, so if PCR is indeed helpful to identify PD-L1 non-expressors, its use may avoid unnecessary IHC tests. The reliability of this approach remains to be determined in subsequent studies. Overall, the CLOVER investigation calls for further efforts related to the harmonization of PD-L1 testing. Contrary to many studies, the CLOVER considered PD-L1 RNA measurement as a categorical variable by grouping tumors as “positive” and “negative” (37). It is essential to consider RNA expression as a continuous variable. Furthermore, the estimation of meaningful thresholds requires tedious consideration of various clinical and laboratory endpoints. The CLOVER study utilized a conditional threshold for the PCR test and did not adjust its value; so the additional efforts are needed to define the categories of PD-L1 expressors using biologically or clinically relevant criteria. Most importantly, the CLOVER investigation used IHC tests as a comparator for PCR assays. Ideally, studies of this type should consider treatment outcomes instead of surrogate markers; this is particularly true for PD-L1 testing, given that many PD-L1/PD1 targeted drugs show activity irrespective of the results of PD-L1 analysis (6, 7).

The instances of discordant results of PD-L1 expression measurement deserve a more systematic investigation on a case-by-case basis, given that the outcome of PD-L1 testing may dramatically influence clinical treatment decisions. There are examples of surprising discordance, when the same specimen is strongly positive by one antibody but clearly negative by another IHC assay (38, 39). Several factors may contribute to these discrepancies. Human error may be one of the factors when large series of tumors are analyzed. The Blueprint project revealed that the inter-observer variability may play a role in the interpretation of the results of PD-L1 staining (38). PD-L1 IHC assays calculate only the proportion of stained cells, while the intensity of the staining is not considered; therefore, the cut-off point between weak and absent staining may be defined differently. Intratumoral heterogeneity of PD-L1 expression may also contribute to these discrepancies, given that even serial sections may differ from each other with regard to the percentage of stained cells. The process of industrial development of distinct PD-L1 antibodies by definition involves distinct protein epitopes and distinct animals, so the individual antibody clones may differ in their ability to recognize various PD-L1 isoforms. The incorporation of the RNA testing adds complexity to this issue. It is not impossible that some tumor specimens lose their ability to interact with diagnostic antibodies during the archiving process; these samples may retain detectable PD-L1 RNA expression but show PD-L1 negativity by IHC.

There is a growing enthusiasm towards the use of PCR-based expression assays as a substitute or complement for IHC analysis. For example, Oncotype Dx breast cancer panel includes estrogen receptor, progesterone receptor and HER2 measurement to aid conventional IHC testing (40). Some studies demonstrate that Ki-67 RNA-based expression analysis has non-inferior or even better clinical performance as compared to conventional IHC tests (41, 42). There are several reported PCR-based biomarkers, which could assist the administration of immune checkpoint inhibitors (18, 43). It is highly likely that PD-L1 testing will undergo significant modification in a very near future.

## Data Availability Statement

The original contributions presented in the study are included in the article/supplementary material, further inquiries can be directed to the corresponding author/s.

## Author Contributions

All authors listed have made a substantial, direct and intellectual contribution to the work, and approved it for publication.

## Conflict of Interest

The authors declare that the research was conducted in the absence of any commercial or financial relationships that could be construed as a potential conflict of interest.
